# Secreted GRP78 activates EGFR-SRC-STAT3 signaling and confers the resistance to sorafeinib in HCC cells

**DOI:** 10.18632/oncotarget.15223

**Published:** 2017-02-09

**Authors:** Rui Li, Gu Yanjiao, He Wubin, Wang Yue, Huang Jianhua, Zheng Huachuan, Su Rongjian, Luan Zhidong

**Affiliations:** ^1^ Department of Cell Biology, College of Basic Medicine, Jinzhou Medical University, Jinzhou, China; ^2^ Department of Pathology, College of Basic Medicine, Jinzhou Medical University, The First Affiliated Hospital of Jinzhou Medical University, Jinzhou, China; ^3^ The First Affiliated Hospital of Jinzhou Medical University, Jinzhou, China; ^4^ Department of Cell Biology, College of Basic Medicine, The First Affiliated Hospital of Jinzhou Medical University, Jinzhou, China; ^5^ The First Affiliated Hospital of Jinzhou Medical University, Jinzhou, China; ^6^ Life Science Institute of Jinzhou Medical University, Jinzhou, China; ^7^ Life Science Institute of Jinzhou Medical University, College of Basic Medicine of Jinzhou Medical University, Cell Biology and Genetic Department of Jinzhou Medical University, Key Lab of Molecular and Cellular Biology of the Education Department of Liaoning Province, Jinzhou, China; ^8^ Development Department of Jinzhou Medical University, Life Science Institute of Jinzhou Medical University, Jinzhou, China

**Keywords:** Grp78, sorafenib, EGFR, STAT3, hepatocellular carcinoma

## Abstract

Acquired resistance is a common phenomenon for HCC patients who undergone sorafenib treatment, however the mechanism by which acquired resistance develops remains elusive. In this study, we found that GRP78 could be detected in the serum samples of HCC patients and the conditional medium of multiple HCC cell lines, suggesting that GRP78 is secreted by HCC cells. Further studies showed that secreted GRP78 facilitated the proliferation and inhibited the apoptosis induced by sorafenib both in HCC cell lines and in tumor xenografts. We further found that secreted GRP78 could interact physically with EGFR, therefore activates EGFR signaling pathway. knockdown of EGFR decreased secreted GRP78 induced phosphorylation of SRC and STAT3. By contrast, overexpression of EGFR further enhanced the phosphorylation of SRC and STAT3 induced by secreted GRP78, suggesting the critical role of EGFR in secreted GRP78 conferred resistance to sorafeinib. Moreover, inhibition of SRC by PP2 antagonized the resistance to sorafenib and inhibited the activation of STAT3 conferred by secreted GRP78. Taken together, our results showed that secreted GRP78 could interact with EGFR, activate EGFR-SRC-STAT3 signaling, conferring the resistance to sorafenib.

## INTRODUCTION

Hepatocellular carcinoma (HCC) is the third leading cause of cancer-related death worldwide [1]. Curative treatments such as surgical and locoregional therapies are the preferred treatment methods for HCC patients at early stages and improve the five-year survival rates up to 60–70% in well-selected patients [2]. However, systemic therapy is still one of the important treatment options for the patients diagnosed at advanced stages.

Sorafenib is an oral multikinase inhibitor that inhibits tumor cell proliferation and increases the rate of apoptosis in many human cancers by inhibiting the serine-threonine kinases BRAF and CRAF and the receptor tyrosine kinases vascular endothelial growth factors receptors (VEGFRs) and platelet-derived growth factor receptor β (PDGFR-β) [3]. Until now, sorafenib is still the only FDA approved systemic drug for the treatment of unresectable advanced HCC [4]. Unfortunately, although sorafenib improved overall survival (OS) of HCC patients, acquired resistance to sorafenib in advanced HCC patients is a common phenomenon and limits its clinical application [5].

The glucose-regulated protein 78 (GRP78) is one of the ER stress induced proteins [6] which is overexpressed in many human cancers [7–9] and plays critical roles in the regulation of tumor proliferation, invasion and metastasis [10–11] and acquired resistance to chemotherapies [12–13]. Traditionally GRP78 regarded as an ER resident protein and act as a molecular chaperone in the lumen of ER. Recently, several lines of evidence have demonstrated that GRP78 could be secreted out of the cell under stress condition. Solid tumor cells including prostate, colon [14], melanoma and breast cancer could secret GRP78 into tumor microenvironment and block the anti-angiogenic effect of bortezomib [15], suggesting GRP78 secreted by tumor cells plays an important role in the resistance to antiangiogenic therapy. It has been reported that secreted GRP78 induces the differentiation of bone marrow mesenchymal stem cells (BMSCs) to cancer-associated fibroblasts and maintain the stability of tumor microenvironment [16].

In this study, we showed that GRP78 secreted by HCC cells promotes the proliferation of HCC cells, and confers the resistance to sorafenib in an autocrine or paracrine manner by interacting physically with epidermal cell growth factor receptor (EGFR) and therefore activates EGFR-SRC-STAT3 signaling.

## RESULTS

### GRP78 could be secreted by HCC cells

**Figure 1:**
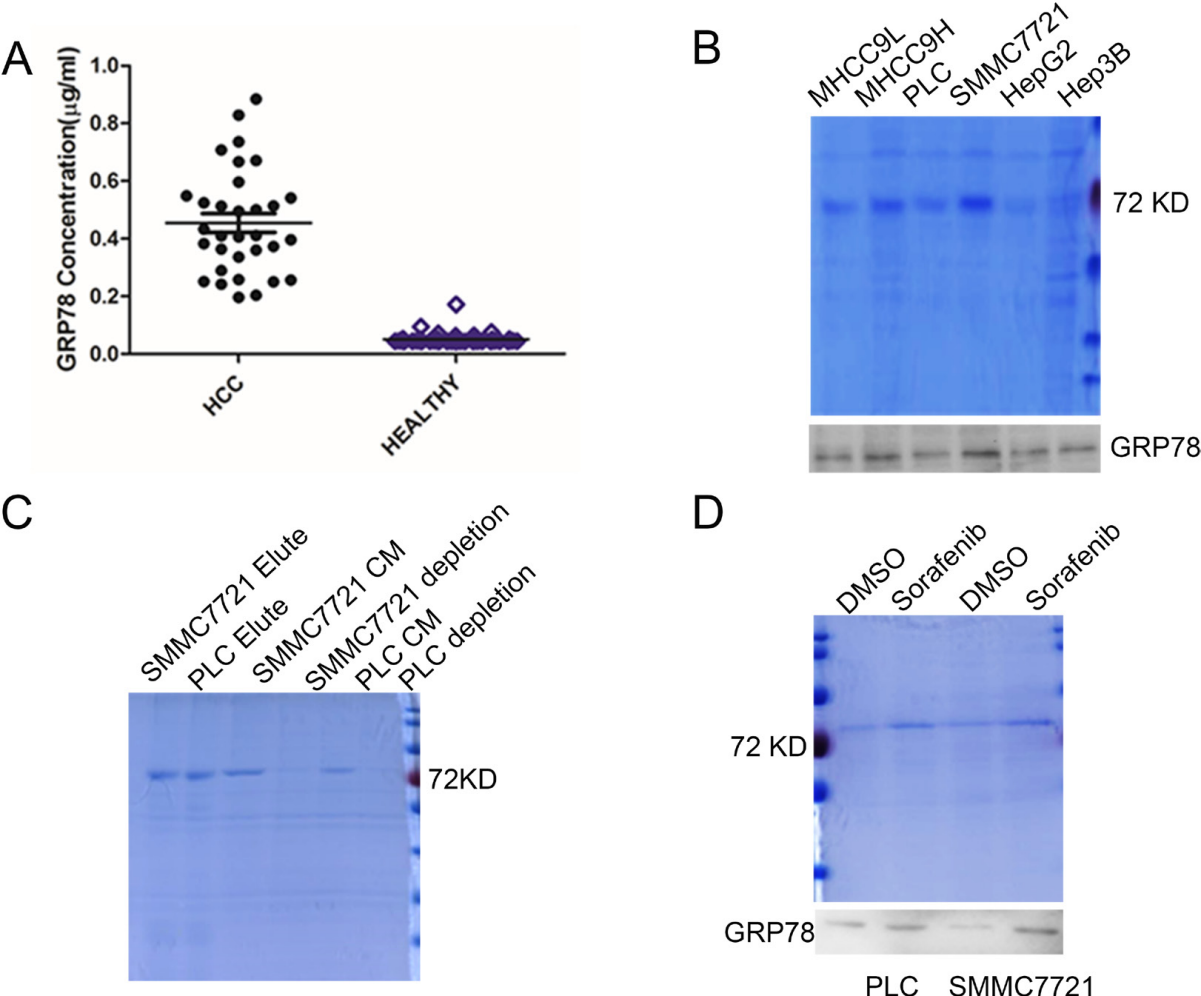
GRP78 is secreted by HCC cells (**A**) ELISA analysis of serum GRP78 in HCC patients. (**B**) SDS-PAGE and western blot analysis of the GRP78 secretion in HCC cells. (**C**) SDS-PAGE analysis of the conditional medium depleted GRP78 using antibody specifically against GRP78. (**D**) SDS-PAGE and western blot analysis of the effect of sorafenib on the secretion of GRP78.

We first examined GRP78 levels in 32 cases of serum samples from HCC patients by competitive enzyme linked immunosorbent assay (ELISA). As shown in Figure 1A, the median value of serum GRP78 in HCC group was ~410.5 ng/ml, indicating a ~10-fold increase as compared with that in control healthy people (~45 ng/ml). Next, we determined whether GRP78 is secreted by HCC cells. Conditional medium(CM) from HepG2, PLC, Hep3B and SMMC7721 cells were condensed to 50% and separated by 10% SDS-PAGE and a protein band whose molecular weight was ~78 KDa was observed. This band was primarily identified as GRP78 by western blot (Figure 1B). The secretion of GRP78 was further confirmed by antibody depletion using antibody specifically against GRP78 in the CM from SMMC7721 and PLC cells. The depleted CM was re-separated by SDS-PAGE and the results revealed that the ~78 KDa protein band was significantly decreased or even disappeared in GRP78 depleted CM as compared with complete CM (Figure 1C). Next, we investigated whether sorafenib could induce the secretion of GRP78 in HCC cell lines PLC and SMMC7721. Serum starved SMMC7721 and PLC cells were treated with 5 μM of sorafenib for 12 h. The secretion of GRP78 was determined using SDS-PAGE and western blot. The results revealed that sorafenib treatment resulted in a significant increase in the secretion of GRP78 as compared with control cell treated with DMSO (Figure 1D).

Taken together, these data suggested that GRP78 could be secreted by HCC cells and the secretion of GRP78 could be further induced when treated with sorafenib.

### Secreted GRP78 facilitates the proliferation and inhibits cell apoptosis induced by sorafenib

**Figure 2:**
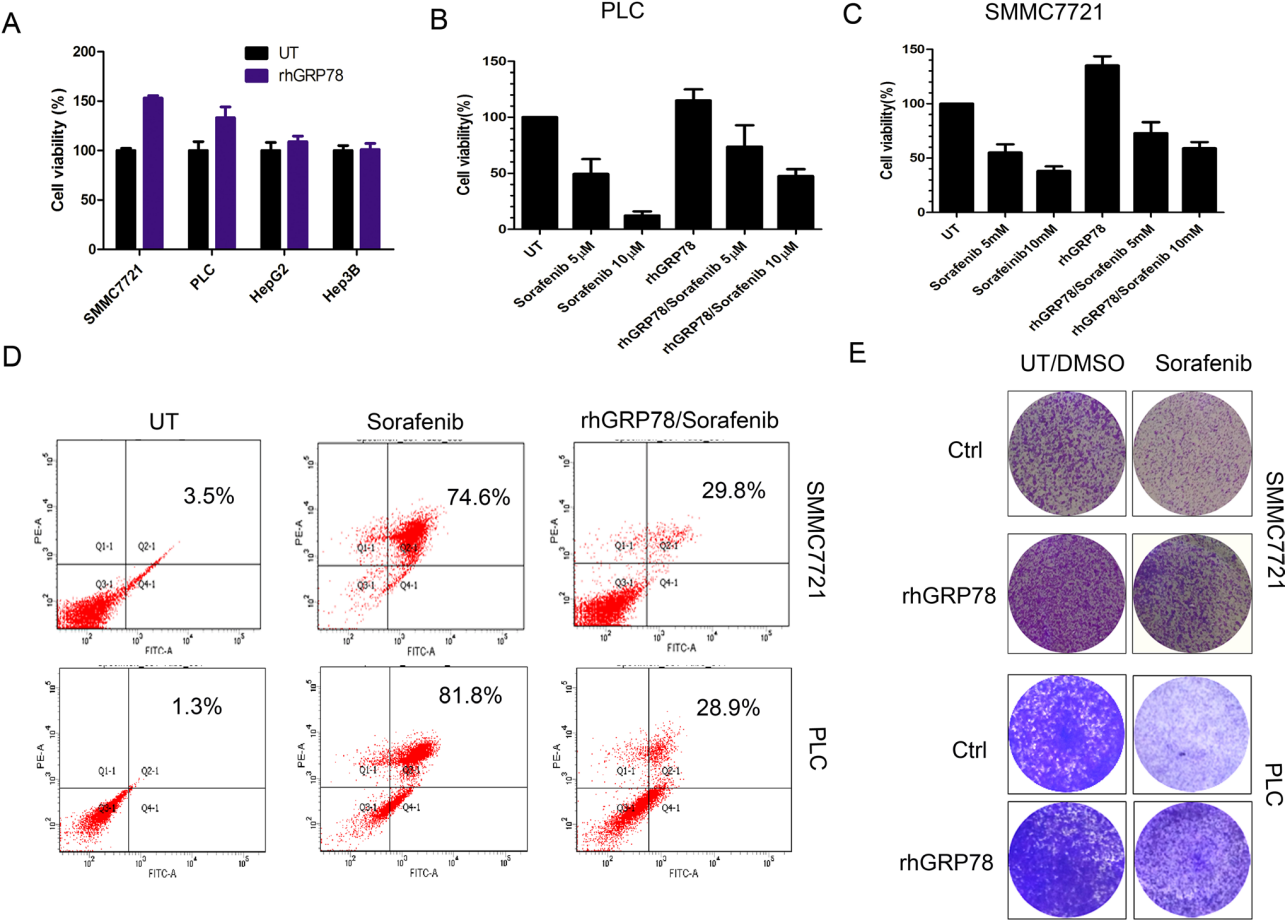
Secreted GRP78 promoted the proliferation of HCC cells and inhibited the sensitivity of HCC cells to sorafenib *in vitro* (**A**) MTT analysis of the effect of secreted GRP78 on the proliferation of HCC cells. The experiment was repeated three times in triplicate. The values were presented as mean ± SD. (**B**, **C**) MTT analysis of the effect of secreted GRP78 on the sensitivity to sorafenib in HCC cells in a short term (48 h). The experiment was repeated three times in triplicate. The values were presented as mean ± SD. (**D**) Flow cytometry analysis of the effect of secreted GRP78 on the apoptosis induced by sorafenib. The status of apoptosis was represented by the percentage of apoptotic cells (%). (**E**) Colony formation analysis of the effect of secreted GRP78 on the proliferation and the sensitivity to sorafenib in HCC cells in a long term (14 days). The experiment was repeated three times in triplicate.

We then examined whether secreted GRP78 promoted the proliferation of HCC cells *in vitro*. For this purpose, HCC cell lines SMMC7721, PLC, HepG2 and Hep3B were serum starved for 4h and subsequently cultured in serum free medium containing 400 ng/ml of recombinant human GRP78 (rhGRP78) for 48 h. It is worth to note that the concentration of rhGRP78 we used in this and the following experiments was 400 ng/ml, which was determined according to serum GRP78 levels of HCC patients. MTT assay revealed that rhGRP78 resulted in a significant increase in cell viability in SMMC7721 cells (~1.53-fold increase in cell viability) and PLC cells (~1.33-fold increase in cell viability) as compared with untreated cells, whereas did not affect HepG2(~1.09- fold increase) and Hep3B cells (~1.01- fold increase) (Figure 2A). The conclusion was further demonstrated by the result that rhGRP78 facilitated the colony formation in SMMC7721 and PLC cells (Figure 2E, column 1). Next, we examined whether secreted GRP78 affected the sensitivity to sorafenib in HCC cell lines PLC and SMMC7721. Moreover, tumor Cells were serum starved for 4 h, pretreated with 400 ng/ml of rhGRP78 for 2 h and subsequently treated with 10 μM of sorafenib for 48 h in serum free medium containing rhGRP78 (400 ng/ml). MTT assay showed that rhGRP78 protein maintained cell viability in the presence of sorafenib in PLC (sorafenib versus rhGRP78/sorafenib: 12.76 ± 3.81% versus 47.62 ± 6.63%) and SMMC7721 cells (sorafenib versus rhGRP78/sorafenib: 38.27 ± 4.35% versus 59.34 ± 5.87%) (Figure 2B, 2C), indicating that secreted GRP78 decreased the sensitivity of HCC cells to sorafenib. The results of colony formation assay further demonstrated this conclusion (Figure 2E, column 2). Finally, we examined whether secreted GRP78 inhibited the apoptosis induced by sorafenib. As shown in Figure 2D, rhGRP78 treatment caused a significant decrease in the percentage of cell apoptosis induced by sorafenib in SMMC7721cells (sorafenib versus rhGRP78/sorafenib: 74.6% versus 29.8%) and PLC cells (sorafenib versus rhGRP78/sorafenib: 81.8% versus 28.9%). These data suggested that secreted GRP78 confers the resistance to sorafenib in HCC cells *in viro*.

We next investigated whether secreted GRP78 decreased the sensitivity of HCC cells to sorafenib *in vivo*. To this end, SMMC7721 cells (10^7^/200 μL) were injected into the subcutaneous space of nude mice. When the size of the xenografts reached ~100 mm^3^, the mice of sorafenib and rhGRP78/sorafenib treated group were treated with sorafenib(30 mg/kg) by intragastric administration twice a week over 14 days. As noted, the mice of rhGRP78/sorafenib treated group were treated with rhGRP78 (400 μg/kg) by intraperitoneal injection one day before sorafenib administration. For rhGRP78 treated group, the mice were treated with rhGRP78 (400 μg/kg) by intraperitoneal injection twice a week.

**Figure 3:**
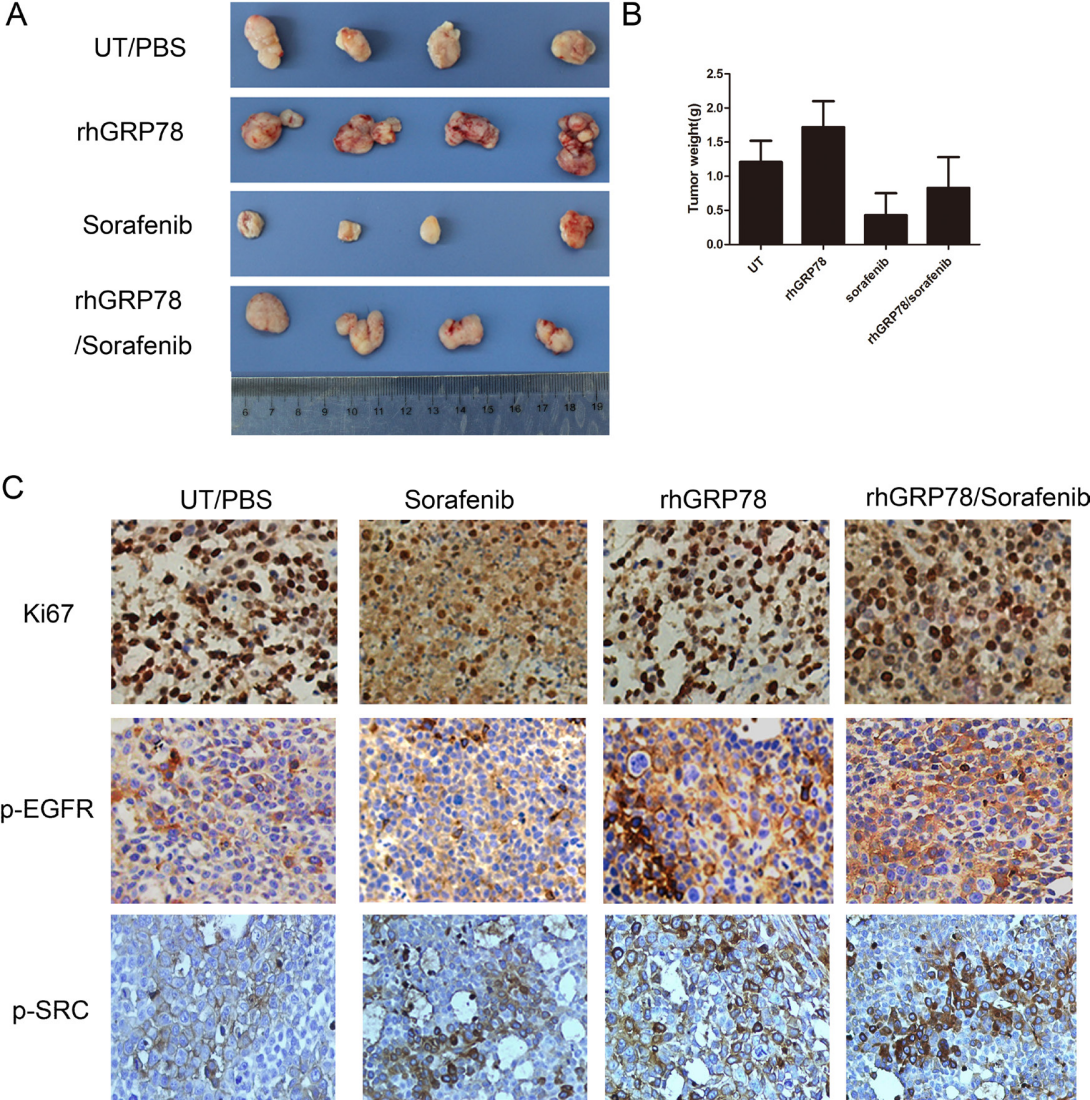
Secreted GRP78 promoted the proliferation of HCC cells and inhibited the sensitivity of HCC cells to sorafenib *in vivo* (**A**) Secreted GRP78 promoted tumor growth and mitigated growth inhibition induced by sorafenib in the xenografts of BALB/C mice (*N* = 5 for each group). (**B**) Quantification analysis of the xenograft weights from mice treated with rhGRP78, sorafenib and rhGRP78/sorafenib (unit: g). (**C**) IHC analysis of the expression of Ki67, p-EGFR and p-SRC in the xenografts from mice treated with rhGRP78, sorafenib and rhGRP78/sorafenib. (Scale bar: 200 μM).

When examining whether rhGRP78 promoted the proliferation of HCC cells *in vivo*, we noted that treatment of tumors with rhGRP78 caused a significant increase in the weight of xenograft (UT versus rhGRP78:1.21 ± 0.31 g versus 1.72 ± 0.38 g), indicating that secreted GRP78 promoted the proliferation of HCC. When comparing the weight of xenografts from sorafeinib treated and rhGRP78/sorafenib treated mice, we found that the mean weight in rhGRP78/sorafenib treated mice was significantly higher than that in sorafenib treated mice (rhGRP78/sorafenib versus sorafenib: 0.83 ± 0.45 g versus 0.43 ± 0.32 g), indicating that secreted GRP78 decreased the sensitivity of HCC cells to sorafenib *in vivo* (Figure 3A, 3B).

Furthermore, we examined the expression of ki-67 in the xenografts by immunohistochemistry (IHC). IHC assay showed increased expression of ki-67 in the xenografts from rhGRP78 treated mice as compared with those from untreated mice. Moreover, the expression of ki-67 in the xenografts from rhGRP78/sorafenib treated group was significantly higher than that in sorafenib treated group (Figure 3C).

Taken together, these data suggested that secreted GRP78 confers the resistance to sorafenib *in vitro* and *in vivo*.

### Secreted GRP78 interacted physically with EGFR and activated the EGFR-SRC-STAT3 signaling

**Figure 4:**
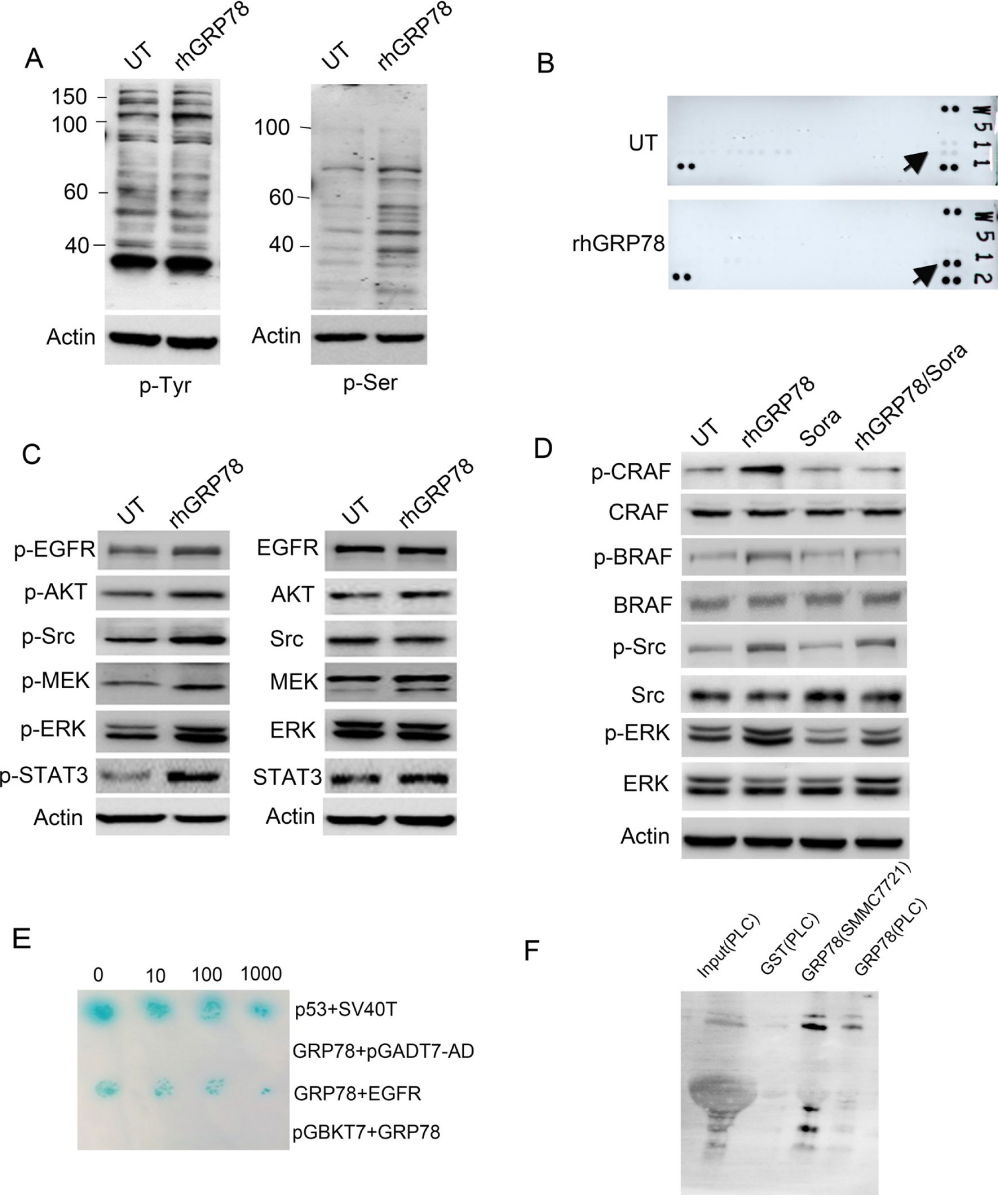
Secreted GRP78 interacted physically with EGFR and activated EGFR downstream signaling (**A**) Western blot analysis of the effect of secreted GRP78 on the phosphorylation of tyrosine and serine in SMMC7721 cells. (**B**) phospho-RTK screening of the cell surface receptor tyrosine kinases in SMMC7721 cells treated with rhGRP78. (**C**) Western blot analysis of the phosphorylation status of EGFR downstream signaling molecules including AKT, SRC, STAT3, MEK and ERK. (**D**) Western blot analysis of the phosphorylation status of BRAF, CRAF, SRC and ERK in SMMC7721 cells treated with rhGRP78, sorafeinib, rhGRP78/sorafenib. (**E**) Yeast two-hybridization screening of the binding partners on the cell surface of SMMC7721 cells using GRP78 as the bait. p53/SV40T was used as the positive control. (**F**) GST pulldown analysis of the interaction between secreted GRP78 and EGFR using rhGRP78 as the bait.

We then tried to explore the molecular basis by which secreted GRP78 conferred the resistance to sorafenib. Serum starved SMMC7721 cells were treated with rhGRP78 for 48 h and the phosphorylation levels of tyrosine and serine were examined by western blot using the antibodies specifically against phospho-Tyr and phospho-Ser. We found a pronounced increase in tyrosine and serine phosphorylation of multiple protein bands, indicating that secreted GRP78 could facilitate the phosphorylation of tyrosine and serine-threonine kinases in HCC cells (Figure 4A). Next, using a phospho-activated antibody array against tyrosine kinase receptors, we found that EGFR had the greatest increase in tyrosine phosphorylation in SMMC7721 cells when treated with rhGRP78 (Figure 4B). Based on these results, we further examined the phosphorylation of EGFR downstream signaling molecules including Src, STAT3, Akt, MEK and ERK by western blot and found that the phosphorylation of these molecules in rhGRP78 treated cells was significantly enhanced as compared with that in untreated cells (Figure 4C).

For sorafenib is a multi-kinases inhibitor, which inhibits the activity of CRAF (RAF1) and BRAF, we next investigated whether secreted GRP78 could antagonize the inhibitory effect of sorafenib on CRAF and BRAF. Western blot analysis revealed that sorafenib treatment caused significant inhibition in the phosphorylation of CRAF and BRAF in SMMC7721 cells treated or untreated with rhGRP78, however maintained the phosphorylation of ERK, indicating the resistance to sorafenib conferred by secreted GRP78 could not be attributed to CRAF and BRAF and there existed an alternative mechanism (Figure 4D).

To explore how secreted GRP78 activated EGFR signaling pathway. We tried to identify cell surface protein that could bind with secreted GRP78. To determine this, two-hybrid (Y2H) screening was performed using GST-rhGRP78 as the bait in human fetal liver cDNA library. The interaction between GRP78 and EGFR was determined by colony formation on yeast SD-Leu-Trp-His-Aba (SD-4) selection media and plate assays for β-galactosidase activity (Figure 4E). Next, GST-pulldown assay was employed using rhGRP78 as the bait to confirm the interaction between GRP78 and EGFR in SMMC7721 and PLC cells. The results revealed the presence of EGFR in the precipitations of PLC and SMMC7721 cells (Figure 4F).

We further investigated the phosphorylation of EGFR, SRC and STAT3 in the xenografts from UT, rhGRP78, sorafenib and rhGRP78/sorafenib treated mice. As shown in Figure 3C, rhGRP78 treatment significantly promoted the phosphorylation of EGFR and SRC in rhGRP78 and rhGRP78/sorafenib treated mice.

Taken together, these data demonstrated that secreted GRP78 directly binds with EGFR, activating of EGFR signaling pathway.

### EGFR decides the responsiveness of HCC cells to secreted GRP78

**Figure 5:**
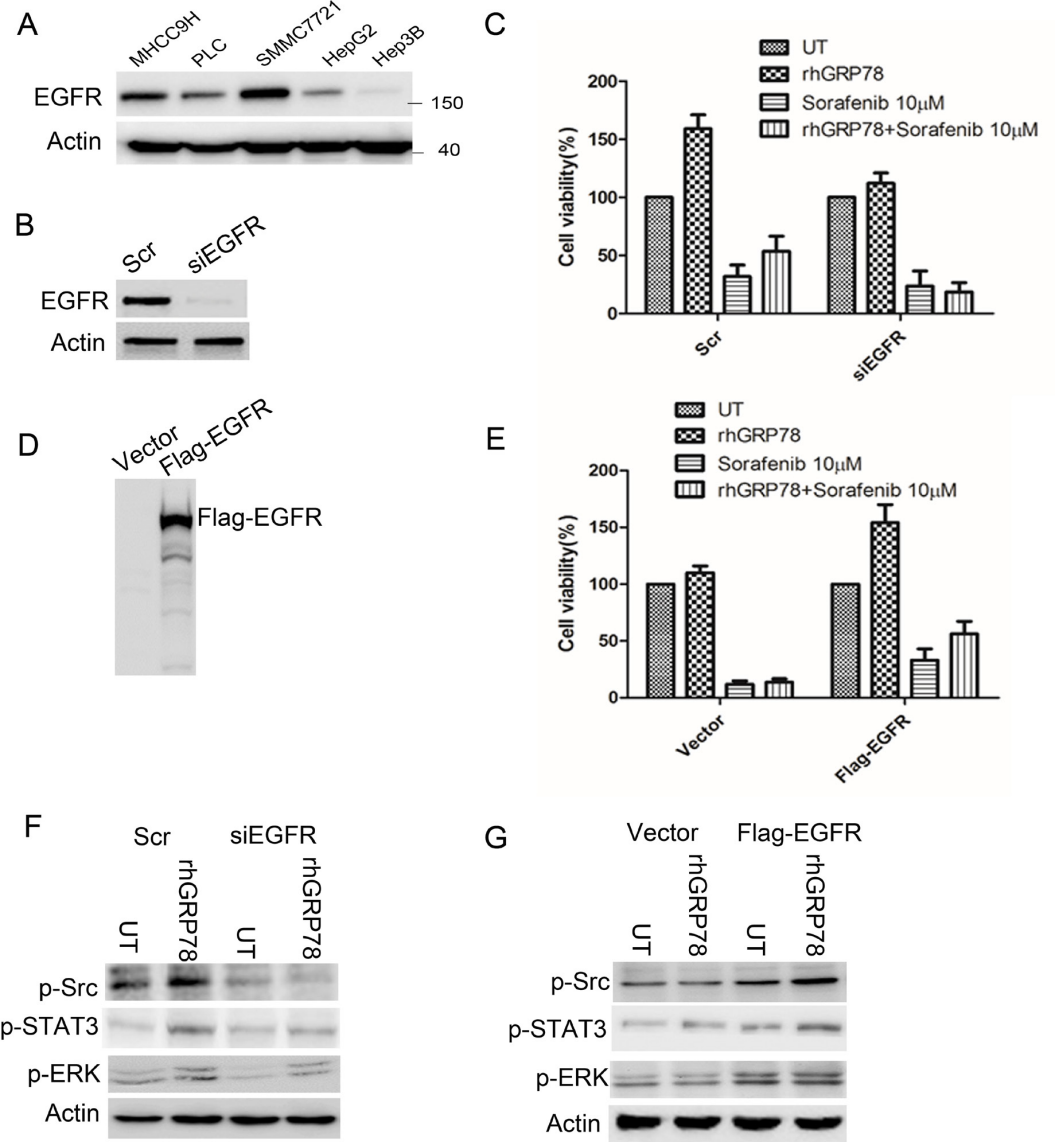
EGFR determines the response to sorafenib in HCC cells (**A**) Western blot analysis of EGFR expression in a variety of HCC cell lines. (**B**) Western blot analysis of EGFR expression in SMMC7721 cells transfected with siEGFR. (**C**) MTT analysis of the effect of rhGRP78 on the sensitivity to sorafenib in EGFR knockdown SMMC7721 cells. The experiment was repeated three times in triplicate. The values were presented as mean ± SD. (**D**). Western blot analysis of EGFR expression in Hep3B cells transfected with Flag-EGFR. (**E**) MTT analysis of the effect of rhGRP78 on the sensitivity to sorafenib in Hep3B cells overexpressing EGFR. The experiment was repeated three times in triplicate. The values were presented as mean ± SD. (**F**) Western blot analysis of the phosphorylation of SRC and ERK in EGFR knockdown SMMC7721 cells. (**G**) Western blot analysis of the phosphorylation of SRC and ERK in cells overexpressing EGFR.

To choose the suitable HCC cell lines for further functional analysis, we examined the expression of EGFR in HCC cell lines. Western blot analysis showed that EGFR was highly expressed in SMMC7721 and MHCC9H cells, moderately expressed in PLC cells, expressed at lower level in HepG2 cells and even no detectable in Hep3B cells (Figure 5A).

Next, we knocked-down EGFR using siRNA in SMMC7721 cells (Figure 5B) and compared the cytotoxic effect of sorafenib on EGFR knockdown and corresponding control cells in the presence of rhGRP78. MTT assay showed that sorafenib treatment resulted in a similar decrease in cell viability in EGFR knockdown cells in the presence and absence of rhGRP78, indicating that secreted GRP78 could not affect the responsiveness of HCC cells to sorafenib under the condition of EGFR knockdown (Figure 5C). Furthermore, we overexpressed EGFR in Hep3B cells by transfection of Flag-EGFR (Figure 5D) and found that overexpression of EGFR caused an obvious increase in cell viability in Hep3B cells when sequentially treated with rhGRP78 and sorafenib as compared with treated with sorafenib alone (Figure 5E). These data suggested that EGFR may decide the responsiveness of HCC cells to secreted GRP78.

Furthermore, we investigated whether rhGRP78 could activate EGFR signaling equivalently in EGFR knockdown SMMC7721 cells by western blot. We found that rhGRP78 treatment had little effect on the phosphorylation of SRC, STAT3 and ERK in EGFR knockdown cells as compared with those in untreated cells (Figure 5F). By contrast, rhGRP78 treatment promoted the phosphorylation of SRC, STAT3 and ERK as compared with untreated cells in Hep3B-EGFR cells (Figure 5G).

**Figure 6:**
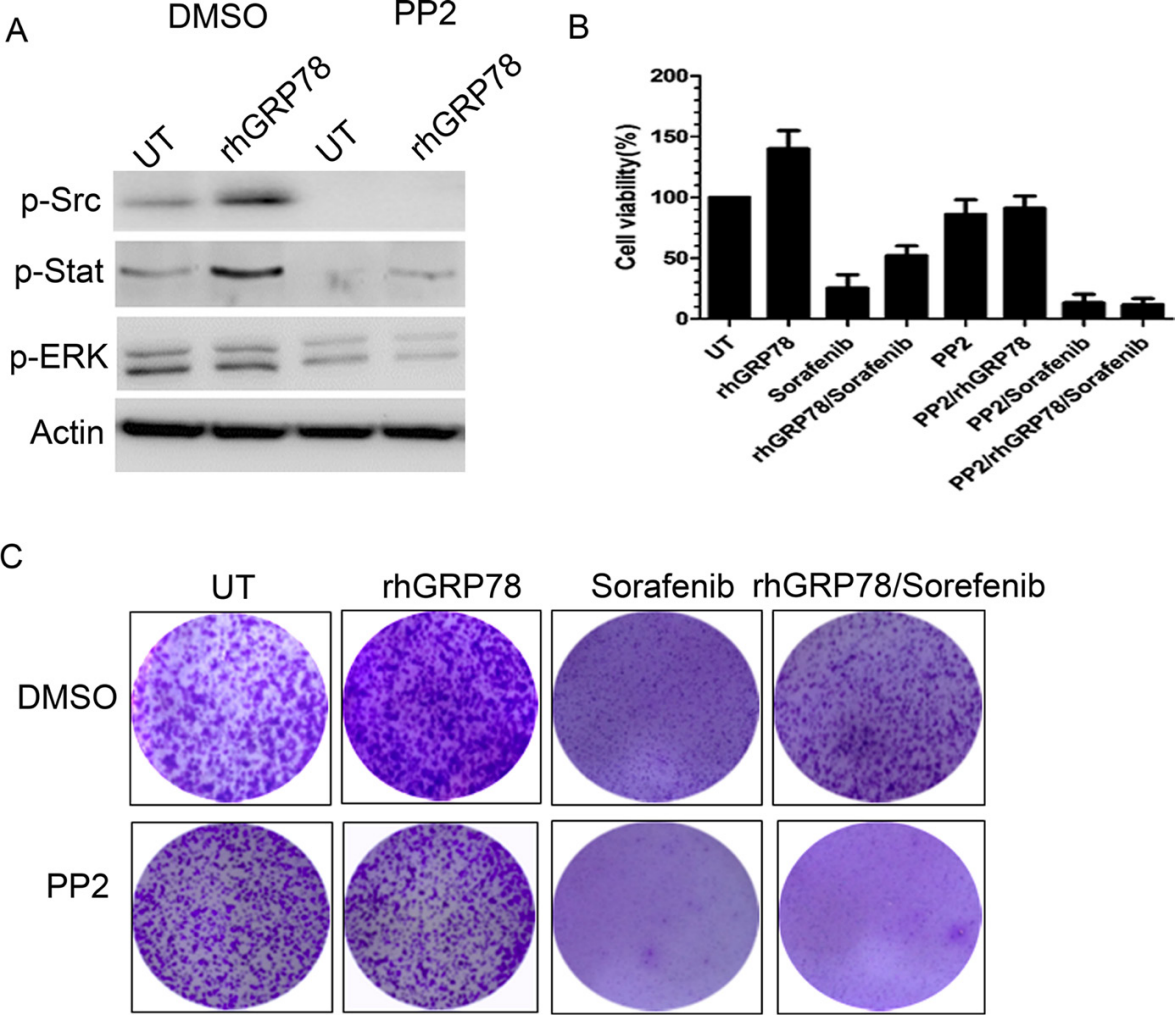
Inhibition of SRC activity attenuates the resistance to sorafenib induced by secreted GRP78 (**A**) Western blot analysis of the effect of PP2 pretreatment on the phosphorylation of STAT3 and EGFR induced by rhGRP78. (**B**) MTT analysis of the effect of PP2 pretreatment on the sensitivity to sorafenib in SMMC7721 cells treated with rhGRP78 in a short term (48 h). The experiment was repeated three times in triplicate. The values were presented as mean ± SD. (**C**) Colony formation analysis of the effect of PP2 pretreatment on the sensitivity to sorafenib in SMMC7721 cells treated with rhGRP78 in a long term (72 h). The experiment was repeated three times in triplicate.

Furthermore, we investigate whether SRC enhanced the phosphorylation of STAT3 in HCC cells. To this end, serum starved SMMC7721 cells were pretreated with PP2, a SRC specific inhibitor, for 1h, followed by rhGRP78 treatment for 12 h in serum free condition. Western blot analysis revealed that treatment of SMMC7721 cells with PP2 resulted in an obvious inhibition in the phosphorylation of STAT3 induced by rhGRP78 (Figure 6A). At the same time, we examined whether inhibition of SRC could attenuate the resistance to sorafenib conferred by secreted GRP78. MTT assay revealed that pretreatment of SMMC7721 cells with PP2 caused a significant increase in the sensitivity to sorafenib in SMMC7721 cells treated with rhGRP78 (Figure 6B). Colony-formation assay also demonstrated that inhibition of SRC significantly increased the cytotoxic effect of sorafenib on rhGRP78 treated SMMC7721 cells (Figure 6C).

## DISCUSSION

Acquired resistance is a common phenomenon for HCC patients undergone sorafenib based regimens and constrains its clinical application [17–20]. The mechanisms by which the resistance develops has been one of the hot issues for current research. Traditionally, GRP78 is regarded as an ER lumen resident protein. Recent advances have indicated that GRP78 can be detected in cytosol, cell surface, nucleus, mitochondria and even be secreted into extracellular environment in many human solid tumors [21–22]. In this study, we tried to investigate whether GRP78 secreted by HCC cells plays a role in the development of sorafenib resistance, if so, explore the mechanism by which secreted GRP78 regulated the sensitivity to sorafenib in HCC cells.

We first demonstrated that GRP78 could be detectable in the serum samples of HCC patients and serum GRP78 level in HCC patients is significantly higher than that in healthy people and correlated with the incurrence of HCC, suggesting that serum GRP78 may serve as a tumor marker in HCC. Similar results were obtained in other solid tumors. For example, serum GRP78 was highly enriched in the serum samples of late-stage lung cancer patients and may be regarded as a prognostic marker for non-small cell lung cancer. Furthermore, we found that HCC cells could secrete GRP78 in a panel of five HCC cell lines and the amount of GRP78 secretion could be further induced by sorafenib treatment.

Since GRP78 is enriched in the serum samples of HCC patients and sorafenib treatment could elevated GRP78 secretion in HCC cells, we wonder GRP78 plays a role in the regulation of tumor cell proliferation and the development of sorafenib resistance. As expected, our results revealed that rhGRP78 promoted the proliferation of HCC cells and inhibited the apoptosis induced by sorafenib treatment *in vitro* or *in vivo*. These results suggested an autocrine or paracrine model by which GRP78 regulated the proliferation and the response to sorafenib treatment in HCC cells.

The key to confirm this hypothesis is to identify the cell surface receptor of secreted GRP78. Using two-hybrid (Y2H) screening, we identified EGFR as a binding partner of GRP78. The interaction of GRP78 and EGFR was further confirmed by GST pulldown assay using rhGRP78 as the bait. Furthermore, we found that rhGRP78 treatment facilitated the phosphorylation of EGFR downstream signaling molecules including EGFR, SRC, STAT3, AKT, MEK and ERK in SMMC7721 cells. Furthermore, knockdown of EGFR in SMMC7721 cells abrogated secreted GRP78 induced SRC, STAT3, AKT, ERK activation and aggravated the apoptosis induced by sorafenib, whereas overexpression enhanced secreted GRP78 induced activation of SRC, STAT3, AKT, ERK and mitigated sorafenib induced apoptosis, indicating the critical role of EGFR in this process. Taken together, these data primarily suggested that GRP78 secreted by HCC cells could interacted physically with EGFR, activating EGFR signaling pathway.

Sorafenib is a multiple kinase inhibitor that inhibits cell proliferation and induce cell apoptosis by inhibiting RAF family members BRAF and CRAF. There are two possible means by which secreted GRP78 inhibited the apoptosis of HCC cells induced by sorafenib in HCC cells. On one hand, secreted GRP78 may maintain the phosphorylation of BRAF and CRAF. Our data exclude this possibility because although secreted GRP78 could activate BRAF and CRAF, sorafenib treatment inhibited the phosphorylation of BRAF and CRAF at similar level in the presence or absence of rhGRP78. This conclusion was further demonstrated by the result that sorafenib treatment could not inhibit ERK activation. On the other hand, secreted GRP78 exerted inhibitory effect on cell apoptosis by alternative mechanism. This mechanism seems reasonable because inhibition of SRC kinase activity antagonized the protection role of secreted GRP78 on HCC cells upon sorafenib treatment and inhibited the phosphorylation of STAT3 in HCC cells simultaneously.

In conclusion, HCC cells could develop the resistance to sorafenib in an autocrine or paracrine manner by elevating the secretion of GRP78 when treated with sorafenib. Secreted GRP78 interacted physically with EGFR and initializes EGFR-SRC-STAT3 signaling, conferring the resistance to sorafenib.

## MATERIALS AND METHODS

### Serum samples

All 32 cases of HCC serum samples were obtained from the inpatients of the Department of Gastroenterology of the General Hospital of Chinese Liberation Army. The 44 cases of control serum samples were from healthy people for physical examination. Before specimen collection, we have get the permission of all the people. The procedures were approved by the Ethics Committee of Jinzhou Medical University. The diagnosis of hepatocellular carcinoma was confirmed by histopathology and none of the patients has received chemotherapy or irradiation. 5 mL of peripheral blood was taken from each patient and the serum was separated and stored in small aliquots at −20°C for further analysis.

### Cell culture

Human hepatocellular carcinoma cell lines SMMC7721, PLC, HepG2, Hep3B and Hunh7 were purchased from the Culture Collection of Chinese Academy of Sciences. The cells were propagated in DMEM supplemented with 10% FBS containing 2 mM glutamine, 100 U/ml penicillin, 100 μg/ ml streptomycin at 37°C, 5% CO_2_ −95% O_2_ and passaged every 3–4 days.

### Tumor cell supernatants

10^6^ cells were seeded in a 35 cm^2^ culture dish. For SDS-PAGE analysis, tumor cells were serum-starved for 4 h, subsequently cultured under serum-free condition in the presence or absence of sorafenib for 12 h. The conditional medium(CM) was collected, condensed to 50% of the original volume and stored at −20°C for SDS-PAGE and western blot analysis. For GRP78 depletion, 5 μg of anti-GRP78 was added in 1ml of CM, incubated overnight on a rotator at 4°C. The antigen-antibody complexes were captured by agarose beads conjugated with protein A/G and removed by centrifuge for 5 min at 12,000 g at 4°C. The supernatants were collected, condensed and subjected for SDS-PAGE analysis.

### MTT assay

Tumor cells were cultured at the density of 5,000 cells per well in 96-well tissue culture plate. After 24 h after plating, tumor cells were serum starved for 4 h. To test the effect of rhGRP78 on the proliferation of HCC cells, serum starved tumor cells were treated with 400 ng/ml of rhGRP78 for 48 h. To analyze the effect of rhGRP78 on the sensitivity to sorafenib in HCC cells. Serum starved tumor cells were pretreated with rhGRP78 for 2 h, subsequently treated with sorafenib under serum free condition in the presence of rhGRP78 for 48 h. After 48 h, MTT reagent was added according to the manufacturer's instructions and the absorbance was determined at 570 nm using a microplate reader. Mean values were calculated from three independent experiments.

### Colony formation assay

Cells were seeded into six-well plates (10^4^ cells per well) and allowed to adhere overnight in regular growth media. Cells were then cultured in the absence or presence of rhGRP78/sorafenib as indicated in complete media for two weeks. Growth media with or without rhGRP78/sorafenib was replaced every three days. After two weeks, tumor cells were fixed with methanol (1%) and formaldehyde (1%), stained with 0.5% crystal violet, and photographed by inverted microscope.

### Mice and *in vivo* tumor studies

All animal experiments were approved and supervised by the Animal Care and Use Committees of Jinzhou Medical University. SMMC7721 cells 10^7^ in 200 μL phosphate-buffered saline were injected subcutaneously to both sides of the dorsal midline in nude mice. Once the tumors reached 80–100 mm^3^ at day 7, mice were randomly divided into four groups: (1), control group (2), rhGRP78 treated group. Mice in this group were treated with rhGRP78 (400 μg/Kg) by intraperitoneal injection once every three days for two weeks. (3). Sorafenib treated group. Mice in this group were treateded with sorafenib (30 mg/kg) by intragastric administration once every three days for two weeks. (4), rhGRP78/sorafenib treated group. Mice in this group were pretreated with rhGRP78 rhGRP78 (400 μg/kg) by intraperitoneal injection before the day of sorafenib administration (30 mg/kg) once every three days for 2 weeks. After two weeks, the xenografts were removed, the tumor weights were measured, and the expression of Ki67, p-EGFR, p-SRC were examined by immunostaining.

### Western blot

Preparation of whole cell lysates and western blot analysis was performed as described. The primary antibodies used were anti-GRP78 (sc-1050), anti-actin (sc-1616) (sc-6216) were obtained from Santa Cruz Biotechnology. Anti-ERK (ab17942), anti-p-ERK (ab47339), anti-AKT (ab179463), anti-p-AKT (ab38449) were purchased from Abcam (Cambridge, UK). Anti-EGFR (#3777), anti-p-EGFR (Y1068, #4267), anti-MEK (#4694), anti-p-MEK (S217/211, #3958), anti-STAT3 (#12640), anti-p-STAT3 (Y705, #9145), anti-c-SRC (#2123), anti-p-c-SRC (Y416, #6943) were purchased from Cell signaling (Danver, USA).

### Immunohistochemistry

Immunohistochemistry was performed on the formalinsixed paraffin sections. Briefly, 5 μm sections were dewaxed, rehydrated and incubated in 0.3% (V/V) hydrogen peroxide in PBS (0.01 M, pH 7.6) for 20 min to inactivate endogenous peroxidase. Antigen was retrieved by high pressure for 2 min in citrate buffer (0.01 M sodium citrate, pH 6.0). The sections were then incubated with 1:100 diluted primary antibodies at 4°C overnight, and then stained with HRP/Fab Polymer conjugated secondary antibodies for 30 min at room temperature. The antibodies were revealed by DAB at room temperature for 1 min. The primary antibodies were replaced by PBS as negative control. All sections were examined and scored independently by two investigators without any knowledge of the clinicopathological data of the patients, at least 5 fields were randomly chosen. Expressions of Ki67, p-EGFR and p-SRC were evaluated according to the ratio of positive cells per specimen and staining intensity. The ratio of positive cells per field was evaluated quantitatively and scored as 0 for staining less than 5%, 1 for staining of 5 to 10%, 2 for staining of 10 to 50%, 3 for staining > 50%. Intensity was graded as follows: 1, weak; and 2, strong staining. A total score of 0 to 6 was calculated and the scores were designated as 1 (score: 0–1), 2 (2–4), and 3 (5–6).

### Enzyme linked immunosorbent assay (ELISA)

GRP78 levels in the serum samples of HCC patients were measured by Enzyme linked immunosorbent assay (ELISA) using a commercially available competitive ELISA Kit (GRP78/BiP ELISA kit, Enzo Life Sciences). The performance performed according to the manufacturer's protocol.
